# FLT3: A 35-Year Voyage from Discovery to the Next Generation of Targeted Therapy in AML

**DOI:** 10.3390/cancers17213415

**Published:** 2025-10-23

**Authors:** Maria-Camelia Stancioaica, Daniel Coriu, Gabriel Ghiaur

**Affiliations:** 1 Faculty of Medicine, “Carol Davila” University of Medicine and Pharmacy, 050474 Bucharest, Romania; daniel_coriu@yahoo.com; 2 Department of Hematology and Bone Marrow Transplantation, Fundeni Clinical Institute, 022338 Bucharest, Romania; 3 The Sidney Kimmel Comprehensive Cancer Center at Johns Hopkins, Department of Oncology, Johns Hopkins University School of Medicine, Baltimore, MD 21205, USA

**Keywords:** acute myeloid leukemia, FLT3, FLT3 inhibitors, midostaurin, gilteritinib, quizartinib, drug resistance, minimal residual disease, bone marrow microenvironment

## Abstract

**Simple Summary:**

Protein kinases are fundamental regulators of cellular function, orchestrating proliferation, survival, and differentiation through reversible phosphorylation. Their importance extends beyond normal physiology: dysregulated kinase activity is a defining feature of cancer, where constitutive signaling sustains malignant growth and therapy resistance. Among them, receptor tyrosine kinases occupy a central role by transmitting extracellular cues into potent intracellular programs. One such receptor, FMS-like tyrosine kinase 3 (FLT3), is expressed at high levels in hematopoietic stem and progenitor cells, where it maintains homeostasis and supports immune development, particularly dendritic cell biology. In acute myeloid leukemia (AML), FLT3 mutations—most often internal tandem duplications (ITDs) or tyrosine kinase domain substitutions (TKDs)—constitute some of the most frequent genetic alterations and are strongly associated with poor prognosis. The therapeutic targeting of FLT3 represents a paradigm for translating kinase biology into clinical progress. Decades of preclinical and clinical work culminated in the approval of three potent FLT3 inhibitors—midostaurin, gilteritinib, and quizartinib—that, together with stem cell transplantation, have dramatically improved outcomes for patients with FLT3-mutant AML. Yet, as with many kinase inhibitors, resistance remains a barrier, fueling efforts toward rational combination therapies and measurable residual disease-directed strategies. The FLT3 story illustrates both the promise and the ongoing challenges of kinase inhibition in modern cancer care.

**Abstract:**

FMS-like tyrosine kinase 3 (FLT3) is a crucial regulator of normal hematopoiesis, with high expression in hematopoietic stem and progenitor cells. Beyond its role in stem cell survival and proliferation, FLT3 signaling is essential for immune regulation, particularly dendritic cell differentiation and NK cell expansion. In acute myeloid leukemia (AML), FLT3 mutations—most commonly internal tandem duplications (FLT3-ITD) and tyrosine kinase domain (FLT3-TKD) substitutions—are among the most frequent genetic alterations, driving constitutive activation of proliferative and antiapoptotic pathways and conferring adverse prognosis. The clinical development of FLT3 inhibitors has been a decades-long endeavor. Early multikinase agents established proof-of-concept but were hampered by off-target effects and incomplete efficacy. The subsequent generation of potent and selective inhibitors has transformed outcomes, culminating in FDA approvals of midostaurin, quizartinib, and gilteritinib. Together with allogeneic transplantation, these agents have reshaped the treatment paradigm for FLT3-mutant AML, converting a historically high-risk subset into one with realistic prospects for long-term survival. Despite these advances, challenges remain. Resistance emerges through cell-intrinsic mechanisms such as acquisition of secondary TKD or RAS pathway mutations, metabolic reprogramming, and antiapoptotic shifts, as well as cell-extrinsic mechanisms mediated by the bone marrow microenvironment, including cytokine support, stromal CYP3A4 metabolism, and retinoid inactivation. These pathways sustain measurable residual disease (MRD), the key predictor of relapse. Rational combination strategies and MRD-directed approaches are therefore essential to fully realize the curative potential of FLT3 inhibition.

## 1. Biology of FLT3

FLT3 (Fms-like tyrosine kinase 3), a member of the class III receptor tyrosine kinase family, was first cloned in 1991 by groups studying hematopoietic growth factors [1,2]. Its ligand, FLT3L (FLT3 ligand), was identified two years later [2,3]. Since then, the FLT3–FLT3L axis has been studied extensively in normal and malignant hematopoiesis. To this end, FLT3 mutations are found in roughly 30% of acute myeloid leukemia (AML) cases and, to a lesser extent, in acute lymphoblastic leukemia (ALL), making it an important therapeutic target [1,2].

### 1.1. FLT3 in Hematopoietic Stem Cells (HSCs)

FLT3 signaling is critical for the survival and homeostasis of early hematopoietic progenitors. Under physiological conditions, receptor activation is ligand-dependent and essential for steady-state hematopoiesis. FLT3L is produced mainly by bone marrow stromal elements, including fibroblasts, and by hematopoietic cells of myeloid, B-, and T-cell origin [2].

FLT3L supports the survival and differentiation of hematopoietic stem and progenitor cells (HSPCs), though by itself it exerts only modest proliferative effects in vitro. In combination with other cytokines such as IL-3, IL-6, IL-7, IL-11, IL-12, and stem cell factor (SCF, the ligand for c-Kit), it markedly enhances HSPC expansion [4]. The c-Kit receptor, another class III tyrosine kinase, shares structural and functional similarities with FLT3 and plays a complementary role in stem cell maintenance and self-renewal. Co-activation of both receptors promotes robust progenitor proliferation and supports multilineage differentiation. This cytokine cross-talk may become clinically relevant is relevant since activation of parallel signaling pathways, such as SCF/c-kit or IL-3/JAK-STAT, can sustain leukemic cell survival and contribute to resistance to FLT3 inhibition.

Notably, FLT3L is highly effective in expanding immature CD34+ progenitors and NK cell precursors via IL-15 induction, a property leveraged in murine models of post-chemotherapy marrow reconstitution and hematopoietic stem cell transplantation [5]. In healthy individuals, circulating FLT3L levels are low, but they rise sharply in bone marrow failure syndromes such as Fanconi anemia or aplastic anemia, likely as a compensatory response to progenitor depletion [5].

### 1.2. FLT3 in Immunity

FLT3L is also indispensable for immune system development and homeostasis. In vivo, daily administration of FLT3L to mice results in a broad expansion of hematopoietic progenitors and multiple immune lineages within bone marrow, spleen, and peripheral blood [5]. These include immature B cells, NK cells, and especially dendritic cells (DCs) across a variety of tissues including in lymph nodes, lungs, liver, thymus, and Peyer’s patches. This expansion has been shown to enhance T-cell-mediated antitumor immunity.

In contrast, FLT3L-deficient (Flt3l-/-) mice display profound leukopenia, reduced marrow and lymphoid organ cellularity, and significant depletion of myeloid and B-lymphoid progenitors, NK cells, and DC subsets [5]. Thymic cellularity, hematocrit, and platelet counts remain unaffected, underscoring the selective requirement for FLT3L. FLT3 signaling also drives plasmacytoid (CD11c+ B220+) and conventional (CD11c+ B220-) DC differentiation via activation of transcription factors such as STAT3 and PU.1. Mice lacking STAT3—a key FLT3 effector—exhibit marked depletion of these DC populations [6,7]. Any therapeutic manipulation of FLT3 must therefore take into account its dual impact on leukemic biology and immune competence. Thus, while FLT3 inhibitors influence immune-cell populations and the bone marrow microenvironment, the present review focuses primarily on the development of FLT3 inhibitors in AML. Readers are referred recent studies discussing the immunomodulatory aspects of FLT3 signaling and inhibition [8,9].

### 1.3. FLT3 Signaling

**Figure 1:**
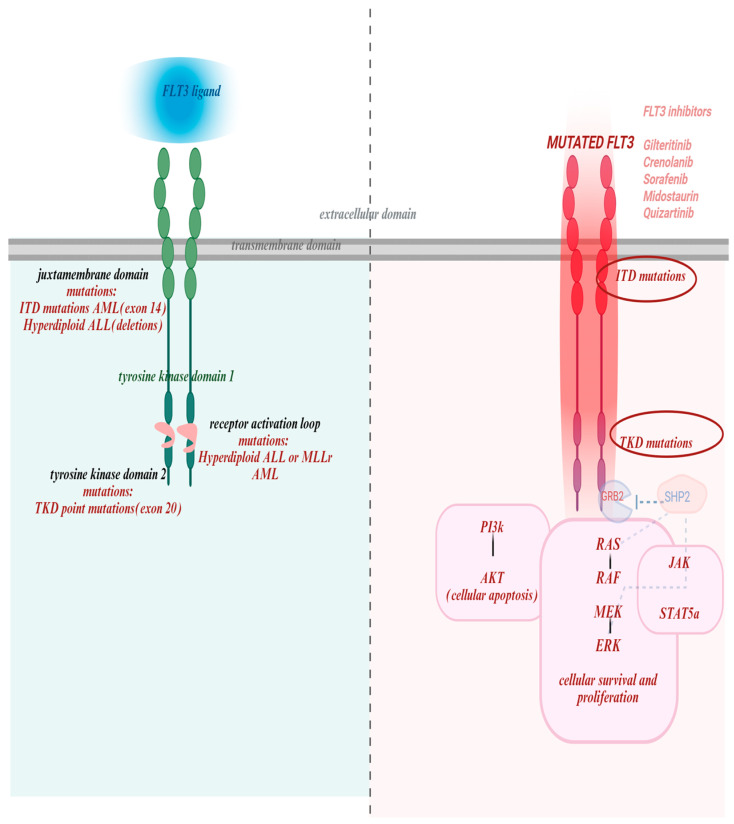
FLT3 structure, mutations and targeted drugs with their binding domains. The left panel illustrates the structure of the wild-type FLT3 receptor, consisting of an extracellular domain that binds FLT3 ligand, a transmembrane domain, juxtamembrane domain and tyrosine kinase domains with the activation domain loop. It also highlights key mutational hotspots: juxtamembrane domain (exon 14): site of internal tandem duplication (ITD) mutations, common in AML, and associated with hyperdiploid ALL (via deletions); Tyrosine kinase domain 2 (exon 20): harbors TKD point mutations, seen in AML; Receptor activation loop: mutations observed in hyperdiploid ALL and MLL-rearranged AML. The right panel pictures mutated FLT3and all of the drugs active against both types of mutations. Also depicts multiple downstream pathways critical for leukemogenesis: STAT5A-signal transducer and activator of transcription 5A; PIK-gene provides instructions for cell growth, division, and survival; SHP2-protein crucial for cell growth, differentiation and movement by regulating the RAS/MAPK signaling pathway [11]. This figure was generated using Biorender (https://www.biorender.com) (accessed on 20 October 2025).

FLT3 activation by FLT3L initiates a cascade of intracellular signaling pathways that regulate cell survival, proliferation, and differentiation [6]. Under normal conditions, receptor activity is tightly regulated and ligand-dependent. Upon ligand binding, the receptor dimerizes and auto phosphorylates, creating docking sites for downstream effectors (Figure 1). As such, binding of FLT3L to FLT3 leads to STAT5A phosphorylation to promote differentiation and immune signaling; activation of PI3K/AKT signaling to support survival and proliferation; and GRB2/SHP2 pathway via phosphorylation of GAB1 and GAB2, which recruit SHP2, GRB2, and PI3K [10].

In vitro, FLT3L acts synergistically with GM-CSF and IL-4 to drive DC differentiation from progenitors. Chimeric receptor studies show that FLT3L plus GM-CSF can transform NIH3T3 fibroblasts and support IL-3-independent proliferation of Ba/F3 hematopoietic cells [10].

c-Kit activation produces overlapping downstream signals, including PI3K/AKT, MAPK, and JAK/STAT, reinforcing the proliferative and survival effects of FLT3. This redundancy means that therapeutic targeting of either receptor requires a nuanced understanding of their distinct and shared contributions to hematopoiesis and immune regulation [12].

Given FLT3’s central role in progenitor biology and immune function, it is perhaps not surprising that aberrations in its signaling have profound implications for malignant transformation. The following section examines FLT3 alterations in hematological malignancies their clinical and therapeutic impact.

## 2. FLT3 in Hematological Malignancies

AML develops as a consequence of accumulation of distinct genetic lesions with cooperative effect. These are broadly classified into class I and class II alterations [2].

Class I mutations, of which FLT3 mutations are a prototypical example, promote proliferation and inhibit apoptosis via constitutive activation of signaling pathways such as STAT5, RAS/MAPK, and PI3K/AKT—Figure 1. Class II mutations, which affect transcription factors or chromatin regulators (e.g., CEBPA, RUNX1, NPM1) lead to impaired differentiation and enhanced self-renewal [2].

### 2.1. FLT3 Mutations in AML

#### 2.1.1. Frequency and Types

FLT3 mutations are detected in ~30% of newly diagnosed AML, 20–40% of APL, and 2–5% of ALL [2,13]. They include internal tandem duplications (ITDs), which insert repetitive sequences and disrupt the juxtamembrane autoinhibitory domain, and tyrosine kinase domain (TKD) point mutations, most commonly at D835, which stabilize the active kinase conformation [14]. By and large, these mutations lead to constitutive activation of the receptor and enhanced signaling via STAT5 and PI3K/AKT pathways in the absence of FLT3L [15].

FLT3-ITDs, present in ~20–25% of adult AML and ~15% in pediatric, are generally associated with more aggressive clinical course and worse prognosis [16,17]. TKD mutations on the other hand, are present in only 5–10% of cases and have a less consistent prognostic impact.

High variant allele frequency (VAF ≥ 0.5) and longer ITDs correlate with inferior survival and increased relapse risk, particularly when NPM1 is not co-mutated. These features are incorporated into European Leukemia Net (ELN) risk stratification [18] [Table 1].

#### 2.1.2. Clinical Presentation

From a clinical standpoint, patients with FLT3-mutant AML often present with marked hyperleukocytosis and high numbers of circulating and bone marrow blasts [19]. Combined with the fact that FLT3-mutant AML, especially with NPM1 co-mutation, tend to have myelomonocytic differentiation (FAB M5), these patients are at high risk of leukostasis and multi organ failure. More so, the high tumor burden, leads to increased incidence of tumor lysis and cytokine release syndrome as well as diffuse intravascular coagulation. In this context, there is characteristic extramedullary involvement, including frequent CNS disease. Thus, CNS prophylaxis should be universally considered for these patients early during treatment.

#### 2.1.3. Prognostic Impact and Co-Mutations

The prognostic impact of FLT3 mutations in AML is increasingly understood to depend on the spectrum of co-occurring mutations [16]. FLT3-ITD mutations frequently co-occur with NPM1 mutations, a pairing that has informed evolving risk stratification systems. In the 2022 European Leukemia Net (ELN) classification, the combination of FLT3-ITD with NPM1 mutation is uniformly considered intermediate risk, irrespective of the FLT3 variant allele frequency (VAF), allowing for more individualized treatment decisions [20]. This marks a departure from the 2017 ELN guidelines, in which FLT3-ITD mutations with high VAF in the absence of NPM1 mutations were classified as adverse risk. More so, FLT3-ITD with low VAF in the presence of NPM1 mutation was classified as favorable risk, which often led clinicians to postpone allogeneic transplantation in first remission (CR1). However, accumulating evidence since then suggests that avoiding transplant under the “favorable” label was often not optimal. Beyond prognosis, NPM1 co-mutation appears to enhance response to FLT3 inhibitors, whereas DNMT3 mutation is linked to treatment resistance and clonal persistence. Of particular interest is the emerging recognition of a so-called “triple-mutant” genotype—FLT3, NPM1, and DNMT3A—which, in the era of FLT3 inhibitors, has been associated with surprisingly favorable outcomes [21]. At the other end of the spectrum, FLT3 mutations are enriched in certain biologically aggressive subtypes of AML, such as those with DEK-NUP214 fusions or KMT2A rearrangements. FLT3 mutations, particularly FLT3ITD are relatively rare in core-binding factor (CBF) AML, including those with RUNX1-RUNX1T1 or CBFB-MYH11 rearrangements and, not surprisingly, they are nearly mutually exclusive with activating mutations in RAS or KIT [16]. As treatment paradigms continue to evolve—particularly with the integration of FLT3 inhibitors—the prognostic significance of co-mutations in FLT3-mutant AML will undoubtedly continue to shift, underscoring the need for dynamic, genotype-informed therapeutic strategies.

#### 2.1.4. Historical Treatment Context

As early as two decades ago, prior to the widespread adoption of FLT3 testing and next-generation sequencing, as most cases of FLT3-mutant AML presented with a normal karyotype, they were therefore classified as intermediate-risk disease. Since the FLT3-mutant clone at diagnosis often comprised only a minor fraction of the total leukemic burden [22], these patients typically achieved initial remission with conventional cytarabine-based chemotherapy. Many of these patients were treated with consolidation chemotherapy alone. Unfortunately, a substantial proportion of these patients relapsed rapidly during consolidation, at which point the disease was uniformly FLT3-mutant and refractory to standard chemotherapy [23]. This clinical course had two major consequences: it led to the early adoption of bone marrow transplantation (BMT) as a standard consolidation strategy in FLT3-mutant AML, and it spurred the accelerated development of FLT3 inhibitors. The incorporation of BMT into the treatment paradigm significantly improved outcomes, with overall survival increasing from approximately 20% in 2005 to nearly 40% by 2011 [17,24]

### 2.2. FLT3 Mutations in APL

FLT3 mutations are also observed in APL, with a reported incidence of approximately 30–40%, comparable to or slightly higher than in non-APL AML [25]. Their presence is strongly associated with high-risk disease by Sanz criteria, primarily due to accompanying hyperleukocytosis [25].

Interestingly, FLT3-mutant APL often exhibits atypical phenotypic features, most notably the microgranular variant morphology in which blasts lack the characteristic coarse granules of classical APL [26]. These cases frequently express CD34 and HLA-DR—markers typically absent in standard APL immunophenotyping—further distinguishing this subset [26]. Clinically, patients with FLT3-mutant APL present with higher rates of disseminated intravascular coagulation (DIC) and early mortality, largely attributable to the elevated white blood cell count at diagnosis [26]. As in non-APL AML, CNS prophylaxis is strongly recommended in this high-risk group [27].

Historically, FLT3 mutations were associated with an increased relapse risk in patients treated with all-trans retinoic acid (ATRA) plus chemotherapy. However, the introduction of ATRA combined with arsenic trioxide (ATO) has largely mitigated this negative prognostic effect, significantly improving outcomes [27]. Nevertheless, recent data suggest that high FLT3-ITD VAF or longer ITD length may still confer some adverse prognostic significance, even in the ATRA/ATO era [28]. Despite these observations, there is currently no established role for FLT3 inhibitors in the treatment of FLT3-mutant APL [Table 1].

### 2.3. FLT3 in Acute Lymphoblastic Leukemia

FLT3 mutations are overall uncommon in ALL compared with AML, and routine clinical testing for FLT3 genetic alterations is not standard practice. Certain genetic subgroups, however, demonstrate higher mutation frequencies. These include MLL-rearranged and hyperdiploid B-ALL, where FLT3 mutations occur in approximately 2–22% of cases, as well as early T-cell precursor (ETP) ALL, in which rates as high as 37.5% have been reported in small adult cohorts [29].

The mutational spectrum in ALL differs from that in AML, with reports of non-canonical variants involving tyrosine kinase domain hotspots (e.g., D835) and the juxtamembrane (JXM) domain, both capable of driving constitutive FLT3 activation [29,30]. FLT3 mutations are also among the kinase-activating lesions identified in Philadelphia chromosome–like (Ph-like) ALL, a high-risk subtype characterized by diverse genetic alterations activating tyrosine kinase signaling [31].

Importantly, while FLT3 mutations are relatively infrequent overall, FLT3 overexpression is nearly universal in ALL [29,32]. Certain subtypes—such as MLL-rearranged, hyperdiploid B-ALL, mixed phenotype acute leukemia, and ZNF384-rearranged ALL—exhibit particularly high expression levels. This overexpression, documented at both RNA and protein levels, can occur independently of FLT3-ITD or TKD mutations and may result from mechanisms such as enhancer hijacking (e.g., 13q12.2 deletions) that upregulate transcription without altering the coding sequence [33] [Table 1].

Clinically, FLT3 overexpression has been associated with adverse prognosis, particularly in MLL-AF4+ B-ALL, where it serves as an independent predictor of poor survival and treatment failure [34,35]. Some studies also suggest that FLT3 mutations may be linked to higher relapse rates, especially in pediatric populations [34]. Functional studies show that blasts with FLT3 mutations or high FLT3 expression exhibit constitutive phosphorylation and are sensitive to FLT3 inhibition, supporting the biological and therapeutic relevance of targeting this pathway. Notably, in relapsed or refractory ALL, FLT3 inhibition may restore chemosensitivity in cases harboring activating mutations, making FLT3 a potentially actionable target in selected patients [34,36].

Collectively, these observations indicate that in ALL, FLT3 abnormalities—whether through mutation or overexpression—are biologically significant and may carry both prognostic and therapeutic implications.

The evolving treatment landscape, aided by the widespread availability of next-generation sequencing, has brought FLT3 alterations into sharper focus across multiple leukemias. This convergence of biologic insight, diagnostic capability, and clinical need laid the foundation for the rapid development of FLT3 inhibitors—a progression explored in detail in the next chapter.

## 3. Development of FLT3 Inhibitors in AML

The clinical development of FLT3 inhibitors has evolved over the last two decades from exploratory off-label use to rigorously designed randomized clinical trials. As mentioned above, in the early 2000s, it became evident that most such patients with FLT3 mutant AML relapsed early, with disease characterized by high variant allele frequency (VAF), dependence on FLT3 signaling, and resistance to chemotherapy. This grim clinical scenario motivated the incorporation of allogeneic BMT into standard of care, while the research community sought pharmacologic approaches to target FLT3. The earliest attempts repurposed existing multikinase inhibitors with incidental FLT3 activity, marking the first chapter in the clinical development of FLT3 inhibitors [Table 2].

Table 2 provides an overview of the main clinical and biological characteristics of approved and investigational FLT3 inhibitors, illustrating the steady refinement of FLT3-directed therapy from early multikinase inhibitors to highly selective agents with defined roles across different stages of AML.

The first-in-class agent midostaurin, a multikinase type I inhibitor, demonstrated a survival benefit when combined with standard chemotherapy in newly diagnosed FLT3-mutated AML, establishing FLT3 inhibition as a validated therapeutic strategy. Gilteritinib, a selective type I inhibitor active against both ITD and TKD mutations, improved overall survival in relapsed/refractory disease and remains the standard of care in this setting. Quizartinib, a type II inhibitor with potent activity against FLT3-ITD but limited efficacy against TKD mutations, recently gained frontline approval following the QuANTUM-First trial, which confirmed its survival benefit when added to intensive chemotherapy. Crenolanib, highly selective type I inhibitor active against both ITD and TKD—including the gatekeeper F691L mutation—is currently in phase III testing and may offer improved breadth of target coverage with minimal off-target toxicity. Sorafenib, an early multikinase type II inhibitor, provided important proof-of-concept for FLT3 inhibition and demonstrated efficacy as post-transplant maintenance, but its broad kinase profile and toxicity have limited its regulatory approval in AML.

### 3.1. First-Generation FLT3 Inhibitors

The earliest efforts relied on existing tyrosine kinase inhibitors (TKIs) that, by chance, also inhibited FLT3. These “first-generation” FLT3 inhibitors shared several characteristics: they were generally broad-spectrum kinase inhibitors, with a wide off-target profile and correspondingly broad—and sometimes dose-limiting—toxicity. Clinically, they revealed an important biological insight: while FLT3 signaling is essential for the survival of circulating blasts in relapsed/refractory FLT3-ITD AML, it is less critical for the survival of bone marrow blasts [37]. Early treatment with these agents often resulted in the rapid clearance of circulating blasts, coupled with a more gradual differentiation of marrow blasts and a striking surge of mature neutrophils into the peripheral blood. This phenomenon closely resembled the differentiation syndrome first described in acute promyelocytic leukemia (APL) patients treated with all-trans retinoic acid (ATRA) in the 1980s [38]. Similar syndromes are now recognized as class effects of targeted therapies against key driver mutations in AML—including FLT3 TKIs in FLT3-mutant AML, IDH inhibitors in IDH-mutant AML, and, more recently, menin inhibitors in KMT2A-rearranged AML [38]. Although the clinical presentation, timing, and incidence vary across these agents, differentiation syndrome remains a potentially lethal complication that requires prompt recognition and management [38].

Another defining limitation of first-generation FLT3 inhibitors was their variable plasma protein binding, which often resulted in a mismatch between plasma drug concentrations and pharmacodynamic activity [39]. This led to the development of the plasma inhibitory activity (PIA) assay as a more reliable surrogate for in vivo target inhibition, which in turn guided dose-finding and optimization of subsequent FLT3 inhibitor generations [39]. Between 15 and 20 years ago, multiple first-generation FLT3 TKIs were evaluated in attempts to rescue patients with relapsed/refractory AML. Among these, the most noteworthy translational and clinical efforts involved sorafenib and midostaurin, which provided foundational lessons for the design of the more potent and selective second-generation agents.

#### 3.1.1. Lessons Learned from the Use Sorafenib

Sorafenib was originally developed as a Raf-1 inhibitor and later approved for the treatment of several solid tumors, including renal cell carcinoma, hepatocellular carcinoma, and thyroid cancer [40]. In addition to its RAF kinase activity, sorafenib also inhibits several receptor tyrosine kinases, including FLT3, KIT, PDGFR, and VEGFR [41,42]. In 2005, sorafenib gained FDA approval for renal cell carcinoma, and its availability through CTEP facilitated widespread translational research. Early investigator-initiated studies tested sorafenib as a single agent in relapsed/refractory (R/R) FLT3-mutated AML, where it demonstrated significant clinical activity. In a phase 1 study of 50 patients with R/R AML, including 28 with FLT3-ITD and 6 with both ITD and TKD mutations, sorafenib monotherapy achieved complete remission (CR) or complete remission with incomplete hematologic recovery (CRi) in 5 patients, with an additional 17 showing significant reduction in bone marrow or peripheral blood blasts [43]. These responses, however, were transient, highlighting the need for combination strategies.

Rational combinations centered on avoiding chemotherapy-induced FLT3 ligand upregulation, which can blunt FLT3 TKI activity. Azacitidine, a hypomethylating agent, does not increase FLT3 ligand levels, providing a rationale for testing azacitidine plus sorafenib. This combination demonstrated promising activity in both R/R FLT3-mutated AML and newly diagnosed patients [44,45]. In frontline settings, the SORAML trial evaluated sorafenib in combination with standard chemotherapy in newly diagnosed AML irrespective of FLT3 mutation status [46]. While sorafenib improved event-free survival (EFS) and relapse-free survival (RFS), overall survival (OS) was not significantly different, and the experimental arm experienced higher rates of grade 3–4 toxicities. Another phase 2 study, ALLG AMLM16, tested sorafenib in newly diagnosed FLT3-ITD AML, but again, no survival benefit was seen overall, though there were signs of improved outcomes in patients with high VAF and those who underwent allo-HCT in first remission [47].

Taken together, sorafenib demonstrated proof-of-concept that FLT3 inhibition can induce clinical responses in R/R AML, and provided foundational lessons for combination approaches. Nonetheless, its broad kinase inhibition, toxicity profile, and lack of consistent survival benefit in randomized trials prevented its regulatory approval in AML.

#### 3.1.2. Upfront Approval: Midostaurin

Midostaurin was initially developed as a protein kinase C (PKC) inhibitor and later found to inhibit FLT3, KIT, VEGFR, and PDGFR [48]. In vitro studies demonstrated that midostaurin induces G0/G1 arrest in FLT3-ITD AML cell lines and G2/M arrest in FLT3–wild-type AML cells, suggesting mutation-specific activity [49]. As a single agent in R/R AML, midostaurin did not produce complete remissions, though it consistently reduced peripheral blast counts [50,51]. This paved the way for testing in combination with chemotherapy.

The pivotal RATIFY trial (CALGB 10603) evaluated midostaurin with standard 7+3 induction and high-dose cytarabine consolidation in newly diagnosed AML patients aged 18–59 with FLT3 mutations (ITD-high, ITD-low, or TKD) [52]. A total of 717 patients were randomized to receive midostaurin or placebo, with those in remission proceeding to maintenance with the study drug. Midostaurin significantly improved overall survival (hazard ratio [HR] for death 0.78, one-sided *p* = 0.009) and event-free survival (HR 0.78, one-sided *p* = 0.002), with consistent benefit across FLT3 subtypes. Notably, early CR rates at 60 days were similar between arms, suggesting that the survival advantage was not due to higher induction response but rather to improved durability of remission, potentially aided by increased allo-BMT rates. The trial established midostaurin as the first FLT3 inhibitor to gain regulatory approval for AML in combination with intensive chemotherapy.

Subgroup analyses further highlighted its activity in FLT3-TKD AML. In patients with NPM1-mutated/FLT3-TKD or CBF-rearranged/FLT3-TKD disease, outcomes were especially favorable. Adverse events were similar between arms, underscoring midostaurin’s tolerability. Small studies of midostaurin with hypomethylating agents did not demonstrate a significant added benefit, underscoring the importance of pairing FLT3 inhibitors with effective cytotoxic backbones [53].

Compared with sorafenib, midostaurin’s randomized data demonstrated a survival advantage that directly led to approval, while sorafenib remained investigational. Differences in trial design, execution, and industry support likely explain the divergent regulatory outcomes.

### 3.2. Second-Generation FLT3 Inibitors

The limitations of first-generation FLT3 inhibitors—broad kinase inhibition, off-target toxicities, inconsistent pharmacodynamic activity, and incomplete marrow disease clearance—prompted the rational design of a new wave of compounds with greater potency, selectivity, and clinical tolerability. These second-generation FLT3 inhibitors were developed specifically to target FLT3, with narrower kinase profiles that reduce off-target effects while achieving sustained target inhibition at clinically relevant plasma concentrations. The three most clinically advanced agents in this class are gilteritinib, quizartinib, and crenolanib, each distinguished by its kinase binding mode, activity spectrum, and off-target profile, particularly with regard to c-KIT inhibition. While we focus here on the clinical development of gilteritinib and quizartinib as they achieved FDA approval in FLT3 mutant AML, crenolanib is also in advanced clinical development at this time. 

From a structural standpoint, gilteritinib and crenolanib are type I inhibitors, binding the ATP-binding pocket of FLT3 in its active conformation [54,55].This allows them to inhibit both FLT3-ITD and tyrosine kinase domain (TKD) mutations, including the common resistance-associated D835 substitution [56]. By contrast, quizartinib, like sorafenib, is a type II inhibitor that binds an adjacent hydrophobic region accessible only in the inactive conformation of FLT3 [57]. While type II agents can be highly potent against ITD mutations, they have limited activity against D835 TKD mutations—an important consideration, as type II inhibitor exposure can drive the emergence of in-cis D835 mutations as a mechanism of resistance [58]. Rare TKD substitutions such as N676K or N841I, and particularly gatekeeper mutations like F691L, may confer relative resistance to both type I and type II inhibitors [55,57].

Kinase selectivity also has important clinical implications. Potent inhibition of c-KIT, for example, can exacerbate myelosuppression by impairing normal hematopoietic recovery. However, if a drug’s inhibitory concentration (IC50) for c-KIT is significantly higher than that for FLT3, a therapeutic window exists in which FLT3 can be effectively targeted while minimizing c-KIT–related cytopenias. Equally relevant is the degree of activity against wild-type (WT) FLT3: although inhibition of WT FLT3 may contribute to toxicity in the setting of regenerating marrow, it also raises the possibility of therapeutic benefit in FLT3–wild-type AML, as suggested by early quizartinib data and ongoing trials in this population [49].

In developing these agents, careful attention has been paid not only to their kinase-binding characteristics but also to their pharmacokinetic and pharmacodynamic properties in vivo. Drugs with promising in vitro kinase inhibition profiles must achieve adequate plasma exposure and sustained FLT3 target coverage in patients—ideally quantified via PIA assays—to translate into durable clinical benefit. The distinct molecular and pharmacological features of gilteritinib, quizartinib, and crenolanib have shaped their clinical trajectories, with each agent pursuing complementary therapeutic niches in relapsed/refractory disease, upfront combinations, post-transplant maintenance, and even FLT3–wild-type AML [49].

#### 3.2.1. Relapsed/Refractory AML: Gilteritinib

Gilteritinib is a second-generation, orally bioavailable type I FLT3 inhibitor designed with greater potency and selectivity than first-generation agents. Preclinical studies demonstrated strong activity against both FLT3-ITD and TKD mutations, including the D835 substitution, while sparing c-KIT at clinically relevant concentrations [54,56]. Importantly, gilteritinib also inhibits AXL, an alternative signaling kinase implicated in resistance to FLT3 inhibition, as well as anaplastic lymphoma kinase (ALK) and leukocyte tyrosine kinase (LTK). These features offered both breadth of activity and the potential to overcome resistance mechanisms intrinsic to FLT3-mutant AML.

The first-in-human phase I/II study evaluated gilteritinib in patients with relapsed/refractory AML, most of whom had not been previously exposed to a FLT3 TKI [59]. The trial incorporated PIA assays to confirm target engagement and guide dose selection. Gilteritinib was well tolerated, with manageable toxicities, and demonstrated promising clinical activity. In patients with FLT3 mutations, composite complete remission rates (CR/CRh) exceeded 40%, and responses were seen across ITD and TKD subtypes. These data established gilteritinib as a potent and clinically active FLT3 inhibitor.

The pivotal ADMIRAL trial randomized 371 patients with relapsed/refractory FLT3-mutated AML to gilteritinib or investigator’s choice of salvage chemotherapy [60]. Gilteritinib significantly improved overall survival (median OS 9.3 months vs. 5.6 months; HR 0.64), with higher composite remission rates (34% vs. 15%). Importantly, 63 patients in the gilteritinib arm proceeded to allo-BMT compared with only 19 in the chemotherapy arm, and at the time of publication, 38 patients in the gilteritinib arm remained alive on study versus none in the chemotherapy arm. These durable outcomes underscored gilteritinib’s role as a transformative agent in R/R FLT3-mutated AML, leading to its FDA approval in this setting. It should be noted that most patients in ADMIRAL were FLT3 TKI–naïve, whereas in current practice, many patients receive a FLT3 inhibitor during induction, potentially impacting real-world efficacy.

Subsequent studies have evaluated gilteritinib in frontline therapy. In a phase Ib trial, gilteritinib was combined with standard 7+3 induction and consolidation in newly diagnosed FLT3-mutated AML [61]. The regimen was well tolerated, achieved high composite remission rates, and demonstrated activity in both ITD and TKD subtypes. Notably, gilteritinib was continued during consolidation and into maintenance, providing critical early safety data on long-term use. In older or unfit patients, the phase 3 trial comparing azacitidine plus gilteritinib versus azacitidine alone demonstrated higher response rates with the combination, but no significant overall survival benefit, likely reflecting disease biology and competing risks in this population [62]. Building on preclinical synergy with BCL-2 inhibition, triplet regimens have combined azacitidine, venetoclax, and gilteritinib. A phase 1b/2 trial reported impressive remission rates in both newly diagnosed and relapsed/refractory FLT3-mutated AML, though with high rates of myelosuppression and infectious complications [63]. Similarly, a physician’s choice backbone study confirmed the activity of gilteritinib-based triplets but highlighted the ongoing challenge of balancing efficacy and toxicity, particularly with respect to venetoclax duration [64].

Collectively, gilteritinib exemplifies the advances of second-generation FLT3 inhibition: potent activity against both ITD and TKD mutations, sparing of c-KIT to reduce myelosuppression, and unique AXL inhibition to counter resistance [60]. Its proven survival benefit in R/R AML, coupled with ongoing trials in upfront combinations and maintenance settings, positions gilteritinib as a cornerstone of modern FLT3-directed therapy.

#### 3.2.2. Upfront Approval: Quizartinib

Quizartinib is a potent, selective type II FLT3 inhibitor developed to overcome the limitations of earlier multikinase agents [65]. Preclinical studies established that quizartinib achieves plasma concentrations sufficient to inhibit phospho-FLT3 in both FLT3-ITD–mutated and FLT3–wild-type AML cells, while also inhibiting phospho-STAT5 downstream signaling [66]. However, its type II binding mode renders it relatively inactive against D835 TKD mutations, raising the clinical concern that resistance through emergent TKD substitutions may occur [67]. Quizartinib also inhibits c-KIT, and early pharmacodynamic studies confirmed suppression of phospho-c-KIT at therapeutic doses, which likely contributed to the observed cytopenias during clinical development.

The first-in-human phase I study evaluated quizartinib in relapsed/refractory AML [66]. Responses were most pronounced in FLT3-ITD patients, with composite remission rates approaching 50% at optimal doses. Importantly, reductions in circulating blasts were often rapid, though marrow clearance was more gradual. QTc prolongation emerged as a notable toxicity, requiring careful dose selection and monitoring. Subsequent phase II trials confirmed these findings, demonstrating consistent activity in FLT3-ITD AML, with higher response rates in TKI-naïve patients [68,69]. Dose optimization settled on 60 mg daily as the best balance between efficacy, cytopenias, and QTc prolongation. Responses in FLT3–wild-type AML were less frequent, though some activity was observed, suggesting possible contribution from WT FLT3 inhibition.

The phase III QuANTUM-R trial randomized 367 patients with R/R FLT3-ITD AML to quizartinib or investigator’s choice of salvage chemotherapy [70]. Quizartinib achieved a statistically significant improvement in overall survival (median OS 6.2 months vs. 4.7 months; HR 0.76), as well as higher remission rates (48% vs. 27%). Interestingly, benefit was observed even in patients with low FLT3-ITD allelic burden, suggesting that WT FLT3 inhibition may contribute to efficacy. Despite this, regulatory approval was not granted in the United States or Europe, with concerns raised about the modest absolute survival benefit, toxicity profile, and trial design. By contrast, the ADMIRAL trial with gilteritinib demonstrated more robust survival gains and established a new standard for R/R FLT3-mutated AML.

More recently, the QuANTUM-First trial evaluated quizartinib combined with standard 7+3 induction and consolidation in newly diagnosed FLT3-ITD AML [71]. The addition of quizartinib significantly improved overall survival compared to chemotherapy alone (median OS 31.9 vs. 15.1 months; HR 0.78), establishing quizartinib as the first second generation FLT3 inhibitor to gain frontline approval in this setting. Subgroup analyses suggested consistent benefit across ITD burden levels and cytogenetic risk groups. Importantly, quizartinib was administered not only during induction and consolidation but also as continuation maintenance therapy, providing early prospective safety data for long-term administration.

In patients unfit for intensive chemotherapy, quizartinib has been tested in combination with hypomethylating agents or low-dose cytarabine [72]. The regimens demonstrated clinical activity, with response rates around 40–50%, though cytopenias were common, likely reflecting combined c-KIT and WT FLT3 inhibition. Triplet regimens combining quizartinib, hypomethylating agents, and venetoclax reported encouraging response rates, but with high myelosuppressive toxicity, underscoring the ongoing challenge of optimizing dosing and duration [73].

Taken together, quizartinib has shown potent clinical activity in FLT3-ITD AML, validated by survival benefit in both relapsed/refractory and frontline settings. Its type II inhibitor profile, while highly effective against ITD mutations, leaves it vulnerable to TKD-mediated resistance. Nevertheless, activity against WT FLT3 raises the possibility of benefit in non–FLT3-mutant AML, a question under investigation in the QuANTUM-Wild trial. Quizartinib’s approval in the frontline FLT3-ITD setting marks a major milestone, though questions remain regarding optimal combination partners, resistance mechanisms, and long-term tolerability [74].

#### 3.2.3. Crenolanib: The Next Generation of Type I FLT3 Inhibition

Crenolanib is a potent and highly selective type I FLT3 inhibitor that binds the active conformation of the kinase, maintaining activity against both FLT3-ITD and TKD mutations, including the common resistance-associated D835 and the gatekeeper F691L substitutions [55,57]. This dual inhibitory profile distinguishes crenolanib from type II inhibitors such as quizartinib, which lose potency against TKD mutations. In preclinical models, crenolanib produced sustained FLT3 inhibition, suppressed downstream STAT5 signaling, and induced strong cytotoxic effects in both FLT3-ITD and FLT3-TKD AML cells, while sparing c-KIT at therapeutically relevant concentrations [57]. Pharmacokinetically, crenolanib is characterized by rapid absorption, a relatively short plasma half-life of about eight hours, and minimal plasma protein binding, allowing for consistent target coverage with thrice-daily dosing [75].

Early-phase clinical studies confirmed crenolanib’s tolerability and antileukemic activity across various patient populations. In a phase II studies of relapsed or refractory FLT3-mutated AML (NCT01522469, NCT01657682), crenolanib achieved composite remission rates of approximately 37–39% in FLT3-TKI-naive patients, with overall response rates approaching 50% [76,77]. Notably, responses were also observed in patients previously exposed to other FLT3 inhibitors, although at lower frequency.

When combined with standard 7+3 induction chemotherapy in newly diagnosed FLT3-mutated AML, crenolanib produced high composite remission rates—up to 85%—and encouraging early survival outcomes, with manageable gastrointestinal and hepatic toxicities (NCT02283177) [78]. Building on these results, the ongoing phase III trial NCT03258931 is comparing crenolanib plus standard chemotherapy with midostaurin plus chemotherapy in newly diagnosed FLT3-mutated AML, with overall survival as the primary endpoint [78]. Additional studies are exploring crenolanib in relapsed/refractory settings and in combination with hypomethylating agents or venetoclax-based regimens.

Unlike gilteritinib and quizartinib, whose clinical trajectories have centered on relapsed/refractory disease, crenolanib’s development has focused primarily on frontline therapy and prevention of resistance through comprehensive inhibition of both ITD and TKD variants. Its minimal off-target kinase activity may offer advantages in tolerability and hematopoietic recovery. Should the ongoing phase III trials validate its early efficacy signals, crenolanib could emerge as the third FLT3 inhibitor to achieve regulatory approval and may redefine the standard of care in frontline FLT3-mutated AML.

### 3.3. Use of FLT3 Inhibitors in Maintenance

The final step in the clinical development of targeted therapies often involves their evaluation as maintenance strategies, a setting in which the therapeutic index must be especially favorable. In contrast to relapsed/refractory disease—where safety thresholds are lower—and frontline induction, where efficacy must be demonstrated despite concomitant chemotherapy, the maintenance setting emphasizes both tolerability and durable disease control. For FLT3 inhibitors, this represents the most rigorous test of safety and efficacy, and to date, no FLT3 inhibitor has received regulatory approval for use in this context. Nevertheless, a series of clinical studies have provided important insight into their potential role.

Early evidence for the feasibility of FLT3 inhibitors in maintenance came indirectly from frontline and relapsed/refractory trials where prolonged post-consolidation or post-transplant therapy was incorporated into trial design. For instance, in the RATIFY trial, patients randomized to midostaurin continued the drug as maintenance for up to one year following consolidation, demonstrating that long-term administration was feasible without prohibitive toxicity [52]. Similarly, in ADMIRAL, gilteritinib was continued as long-term therapy in responders, and 38 patients remained on treatment at the time of publication, compared with none in the chemotherapy arm [79]. These observations, although not designed to address the question of maintenance efficacy, established early proof-of-principle that prolonged FLT3 inhibition was tolerable and could be sustained in remission.

The first randomized evidence for maintenance benefit came from sorafenib. In the SORMAIN trial, 83 patients with FLT3-ITD AML who underwent allo-BMT in first remission were randomized to sorafenib or placebo for up to 24 months [80,81]. The trial demonstrated a striking improvement in relapse-free survival (2-year RFS 85% vs. 53%) and overall survival (2-year OS 90% vs. 66%)55 [80]. Sorafenib was associated with increased rates of chronic GVHD, consistent with preclinical evidence suggesting that FLT3 inhibition may augment graft-versus-leukemia effects [81].A Chinese multicenter trial provided confirmatory evidence, also showing reduced relapse and improved survival with sorafenib maintenance [82]. Importantly, in both studies, patients were TKI-naïve at the time of transplant, reflecting the treatment landscape of the early 2010s.

The RADIUS trial evaluated midostaurin as maintenance following allo-BMT in 60 patients with FLT3-ITD AML [83]. Unlike SORMAIN, RADIUS did not show a statistically significant improvement in relapse-free or overall survival, though there was a numerical reduction in relapse. Interpretation is complicated by the small sample size, open-label design, and the observation that some patients did not achieve sustained FLT3 inhibition as measured by the PIA, suggesting under-dosing may have limited efficacy.

The MORPHO trial [84], randomized 356 patients with FLT3-ITD AML who underwent allo-HCT to gilteritinib or placebo. The primary endpoint, relapse-free survival, was not met in the overall population, though there was a trend favoring gilteritinib (HR 0.68, *p* = 0.0518). Prespecified subgroup analyses revealed that patients who were MRD-positive prior to or after transplant derived significant benefit from gilteritinib maintenance (HR 0.56, *p* = 0.02 and HR 0.4 *p* = 0.01), whereas MRD-negative patients did not [85]. Subsequent analysis demonstrated that conditioning intensity modulated the impact of gilteritinib, with the greatest benefit observed in patients receiving reduced-intensity conditioning [86]. Together, these data underscore the importance of integrating MRD assessment and transplant platform into the interpretation of FLT3 inhibitor maintenance studies. Unfortunately, at present there are no standardized or regulatory-approved MRD assays for FLT3-mutated AML, which complicates efforts to evaluate and optimize FLT3 inhibitor strategies in the maintenance setting.

Collectively, the randomized evidence paints a nuanced picture. Sorafenib maintenance, in TKI-naïve patients, provided robust and reproducible survival benefits across two independent trials. Midostaurin, tested in the smaller RADIUS study, did not clearly improve outcomes, potentially due to limited pharmacodynamic activity. Gilteritinib, evaluated in MORPHO, revealed no overall survival benefit in an unselected population but demonstrated marked efficacy in MRD-positive patients, providing a precision medicine rationale for selective use. These studies highlight that the impact of FLT3 inhibitors in maintenance depends not only on the choice of agent but also on prior TKI exposure, depth of remission at transplant, conditioning regimen, and GVHD dynamics.

Although FLT3-ITD mutations are also detected in pediatric AML, their incidence (typically 5–15%) is lower than in adults, and most dedicated clinical studies have therefore been conducted in adult populations. Pediatric experience with FLT3 inhibition has largely been derived from cooperative group efforts and early-phase trials. The Children’s Oncology Group (COG) incorporated sorafenib and, more recently, gilteritinib into pediatric regimens (e.g., AAML1031, NCT03730012, and NCT05066718), demonstrating biological activity and manageable toxicity profiles in relapsed or refractory FLT3-mutated AML [87,88]. Similar initiatives by the European pediatric AML consortia (e.g., NCT04493138) are ongoing. While early results indicate comparable target engagement to that observed in adults, response durability and long-term outcomes remain under investigation. Continued inclusion of pediatric patients in international cooperative studies will be essential to define the optimal use of FLT3 inhibitors in this population.

In summary, while no FLT3 inhibitor is yet approved for maintenance, the available evidence suggests that sorafenib provides a clear benefit post-transplant in TKI-naïve patients, and gilteritinib may be highly effective in MRD-positive patients or those undergoing reduced-intensity conditioning. The integration of MRD testing into transplant and maintenance strategies, as exemplified by the MORPHO trial, represents the next step in optimizing the use of FLT3 inhibitors to sustain durable remission after curative-intent therapy.

## 4. Mechanisms of Resistance to FLT3 Inhibitors

**Figure 2:**
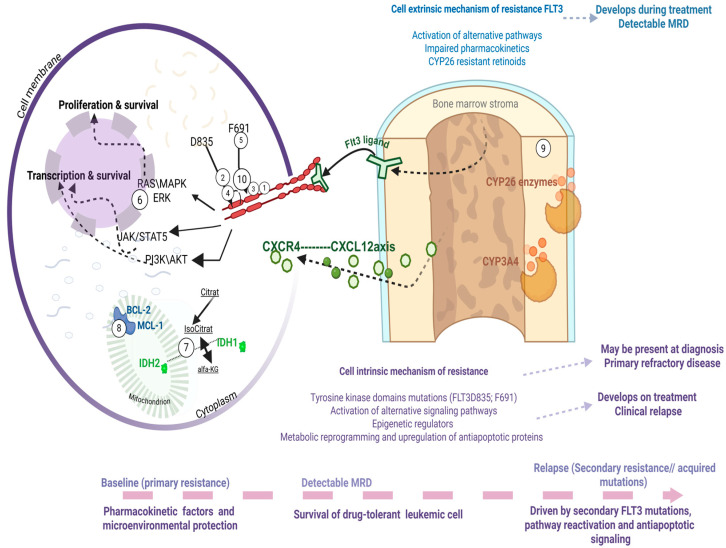
Mechanisms of resistance exemplified with a brief demonstration of intrinsic mechanisms, such as secondary acquisition of mutations, activation of alternative signaling pathways within the cell, alterations in epigenetic regulators, or metabolic changes affecting anti-apoptotic processes. Extrinsic mechanisms of resistance within the bone marrow microenvironment of patients with FLT3 mutations, driven by stromal cell metabolism and local cytokine signaling. Drugs used in clinical trials active against FLT3 mutant AML: Midostaurin (1), Sorafenib (2), Gilteritinib (3), Quizartinib (4), Crenolanib (10). Drugs able to rescue resistance to FLT3 inhibitors: Gilteritinib (3), cellular therapy AMG-553 (5), RAS inhibitor RMC-7977 (6), IDH1 inhibitor Ivosidenib (7), IDH2 inhibitors Enasidenib (7), Venetoclax (8), synthetic retinoids (9), ATO (9). This figure was generated using Biorender (https://www.biorender.com).

The incorporation of FLT3 inhibitors into frontline therapy and the dominant role of gilteritinib in the relapsed/refractory setting have significantly reshaped the landscape of clinical resistance in FLT3-mutant AML. Despite these advances, relapse remains common, and for many patients it represents a fatal event. Thus, clinical resistance to FLT3 inhibitors remains both a pressing unmet need and an area of intense investigation. Conceptually, resistance can be divided into two broad categories: cell-intrinsic mechanisms, arising from genetic, signaling, or metabolic adaptations within leukemic cells, and cell-extrinsic mechanisms, driven by the protective effects of the bone marrow microenvironment and pharmacologic limitations. Both forms of resistance may be present at diagnosis (primary refractory disease) or emerge under therapeutic pressure (relapsed/refractory disease) (Figure 2). Over the last decade, multiple comprehensive reviews have detailed these mechanisms [19]. In this section, we will highlight the most recent insights and emphasize those resistance mechanisms most relevant to ongoing clinical development, while pointing the reader to excellent manuscripts that provide in-depth discussion of specific aspects.

### 4.1. Cell Intrinsic: Acquisition of Additional Mutations

Mechanisms of resistance that arise within leukemic cells themselves are without a doubt central to clinically refractory disease, whether observed upfront or at relapse. These have been reviewed in depth elsewhere [19], and here we focus on concepts of direct clinical relevance.

#### 4.1.1. Tyrosine Kinase Domains Mutations

Mutations within the TKD can be present at diagnosis or emerge under selective pressure. Substitutions in the activation loop (most commonly FLT3 D835) confer resistance to type II inhibitors such as quizartinib and therefore, when present at diagnosis, sway therapeutic decisions toward type I inhibitors like midostaurin [89]. Conversely, mutations in the gatekeeper residue F691 render cells resistant to both type I and type II FLT3 inhibitors [19]. Mutation at this residue alters local conformation, reducing drug binding affinity across FLT3 inhibitor classes. For example, for Quizartinib this is one of the classic resistance known for this drug. Regarding type I inhibitors it is known that Midostaurin it has reduced activity, but not as completely resistant as quizartinib, still efficacy is markedly diminished. For Gilteritinib, F691L it seams to confer substantial loss of sensitivity translated into clinical practice like an acquired resistance mechanism, as for the Crenolanib -in vitro tests show some activity still potency is significantly reduced. Although rare at diagnosis and not typically associated with the same adverse prognosis as FLT3-ITD mutations, these alterations pose formidable clinical challenges when they develop in cis with FLT3-ITD during therapy, as they represent potent mechanisms of acquired resistance that eliminate available inhibitor options [89]. For such patients, the clinical development of cellular therapies such as AMG-553, a FLT3-directed CAR-T cells holds promise, offering a potential strategy to bypass kinase-domain–mediated resistance [90].

#### 4.1.2. Activation of Alternative Signaling Pathways

Another important class of intrinsic resistance mechanisms involves activation of alternative signaling pathways that sustain leukemic cell survival despite effective FLT3 inhibition. Among these, activating mutations in the RAS/MEK/ERK pathway are the most common contributors to primary refractory disease and to relapse after an initial response to FLT3 inhibitors. Efforts to overcome this pathway-driven resistance have focused on the development of direct RAS inhibitors, and most recently, RMC-7977, a pan-RAS inhibitor was used in preclinical studies to provide proof-of-concept that dual targeting may be feasible [91]. Clinical development of RAS inhibitors in myeloid neoplasms is therefore eagerly anticipated.

#### 4.1.3. Epigenetic Regulators

A further layer of complexity is added by epigenetic regulators, which can influence FLT3 inhibitor sensitivity. In particular, IDH1/2 mutations have been associated with relative resistance to FLT3 inhibition. The availability of FDA-approved IDH inhibitors raises compelling questions about optimal sequencing or combination strategies. Phase I trials combining gilteritinib with IDH inhibitors are currently underway [92]. Until then clinicians must often rely on anecdotal evidence and expert consensus when managing patients with co-occurring FLT3 and IDH mutations.

#### 4.1.4. Metabolic Reprogramming and Upregulation of Antiapoptotic Proteins

Metabolic reprogramming and upregulation of antiapoptotic proteins represent important non-genetic mechanisms of resistance. FLT3-mutant blasts under therapeutic pressure can shift their metabolic dependencies and increase reliance on mitochondrial respiration, while simultaneously upregulating antiapoptotic molecules such as BCL-2, MCL-1, and BCL-XL. This biology has clear therapeutic implications: BCL-2 inhibition with venetoclax synergizes with FLT3 inhibitors in preclinical models [93], but clinical application has been challenging due to overlapping myelosuppression and the dynamic balance between BCL-2 and MCL-1 dependence [63,94]. The question of how best to combine FLT3 inhibitors with BCL-2–targeting therapies—whether simultaneously, sequentially, or in rational triplet regimens—remains a key area of ongoing clinical investigation.

### 4.2. Cell Extrinsic: Activation of Alternative Pathways; Impaired Pharmacokinetics; Retinoids

Cell-extrinsic resistance reflects mechanisms driven by the BME and its impact on drug activity, survival signaling, and differentiation dynamics. These mechanisms are clinically relevant not only for FLT3 inhibitors but also for the broader class of targeted therapies in AML, which are similarly vulnerable to cytokine support and stromal metabolism.

#### 4.2.1. General Mechanisms: Cytokine Signaling and Pharmacokinetics

One important contributor is the production of cytokines by marrow stroma, including FLT3 ligand, FGF2, and CXCL12, which sustain alternative pro-survival pathways (RAS/MAPK, PI3K/AKT) in leukemic blasts even in the presence of potent FLT3 inhibition [95]. This phenomenon is particularly evident during cytotoxic chemotherapy, when surges in circulating FLT3 ligand activate the wild-type FLT3 receptor, attenuating the impact of FLT3 TKIs. In this context, the recent development of covalent FLT3 inhibitors such as FF-10101 may eliminate the impact of FLT3 ligands levels on FLT3TKI effectiveness [96,97]. Another critical mechanism is impaired pharmacokinetics: stromal expression of CYP3A4 can metabolize FLT3 inhibitors locally, creating biochemical sanctuaries within the niche [98]. These insights likely extend to other targeted agents, since most novel small molecules in AML are CYP3A4 substrates, and the narrower their selectivity, the more readily AML cells can bypass inhibition by engaging alternative signaling pathways.

#### 4.2.2. Differentiation, FLT3 Expression, and Retinoid Metabolism

A unique aspect of extrinsic resistance in FLT3-mutant AML arises from the fact that mutant FLT3 expression remains under the control of the endogenous promoter. Thus, FLT3 levels decrease as blasts differentiate. This leads to several important observations: (1) AML blasts in the bone marrow, which are less differentiated, express higher levels of FLT3 and are therefore more resistant to inhibition [98]; and (2) as normal myeloid differentiation naturally downregulates FLT3, differentiation therapy represents a rational complement to FLT3 inhibition. Indeed, preclinical studies showed that ATRA synergizes with FLT3 TKIs to eliminate FLT3-mutant leukemia stem cells [99]. However, the BME itself protects blasts from both endogenous and pharmacologic retinoids by expressing CYP26 enzymes, which degrade retinoic acid [100,101].

Recently, this paradigm was advanced by evidence that arsenic trioxide (ATO) downregulates stromal CYP26B1, thereby restoring retinoid signaling within the niche and re-sensitizing FLT3-mutant AML to gilteritinib. In xenograft models, the combination of gilteritinib + ATO prolonged treatment-free survival, a surrogate for MRD reduction, and patient-derived blasts similarly showed improved FLT3 TKI sensitivity in stromal coculture [102]. This provides proof-of-concept that BME-dependent resistance to FLT3 inhibitors can be therapeutically targeted by modulating stromal retinoid metabolism.

Because the persistence of MRD represents the quintessential form of BME-mediated resistance to FLT3 TKIs, and because we now have the tools to measure MRD with high sensitivity, the field is poised to launch MRD-directed interventional trials. These should test whether disrupting niche-mediated protection—through strategies such as differentiation agents, CYP26 inhibition, or ATO combinations—can finally eliminate MRD and reduce relapse risk in FLT3-mutant AML.

## 5. Conclusions

Over three decades of research have transformed FLT3 from a hematopoietic growth factor receptor into one of the most clinically relevant therapeutic targets in AML. The approvals of midostaurin, gilteritinib, and quizartinib have reshaped the prognosis of FLT3-mutant AML, demonstrating that precision targeting of a single receptor tyrosine kinase can alter the natural history of a once uniformly high-risk disease. Yet, the persistence of MRD and the emergence of resistance—through both intrinsic genetic adaptations and extrinsic protection from the bone marrow niche—underscore the limits of FLT3 inhibition as monotherapy. The next phase of this journey will require rational combinations, MRD-guided interventions, and strategies that disrupt microenvironmental support. At the same time, ongoing exploration of FLT3 inhibition in AML without FLT3 mutations and in selected subsets of ALL highlights the broader translational potential of this pathway. Looking ahead, the extension of FLT3-targeted strategies to other hematologic malignancies such as MDS and even to solid tumors with aberrant FLT3 expression, represents an exciting frontier for future investigation. The FLT3 story, therefore, exemplifies both the remarkable progress and the ongoing challenges inherent in targeting kinases in cancer.

## Figures and Tables

**Table 1:** FLT3 mutation in AML, APL, ALL (side-by-side comparison across diseases).

Disease	Frequency of FLT3 Alterations	Predominant Mutation Type(s)	Common Co-Mutations	Clinical Associations	Prognostic Impact	Current Role of FLT3 Inhibitors
AML	~30%	ITD (20–30%) (15% in pediatric population) TKD (5–10%)	NPM1 DNMT3A	Hyperleukocytosis, CNS involvement	VAF & ITD length matter Co-mutations shift risk	Approved and standard of care in some settings
APL	30–40%	ITD, TKD	-	DIC Microgranular variant	Mitigated by ATRA + ATO High VAF/long ITD may still worsen	None established. Salvage therapy in relapsed APL (case reports and Ghiaur personal communication
ALL	2–22% (higher in certain subgroups: 18% of MLLr 28% of hyperdiploid)	ITD, TKD overexpression	MLLr ZNF384r	Poor prognosis in MLL-AF4+ High relapse rates	Overexpression adverse in certain subtypes	Investigational (notably sensitive to FLT3 inhibition)

MLL, rearranged MLL gene; ZNF384 transcription factor gene located on chromosome 12p13, fusions occurs mainly in B-cell precursor ALL; hyperdiploidy (>50 chromosomes); ITD, FLT3 internal tandem duplication; VAF variant allele frequency; ATRA + ATO, All-trans Retinoic Acid + Arsenic Trioxide standard of care for APL.

**Table 2:** Summary of FLT3 Inhibitors and Their Key Properties.

FLT3 Inhibitor	Type & Binding Class	FLT3 Targets	Other Kinase Targets	Key Clinical Trials	Major Limitations	FDA-Approved Indication
**Midostaurin**	Type I (multikinase inhibitor)	FLT3-ITD, FLT3-TKD	PKC KIT PDGFR VEGFR	RATIFY (CALGB 10603, frontline FLT3-mut AML +7+3) RADIUS (post-allo-HCT maintenance)	Multikinase off-targets modest single-agent activity	Approved (2017) for newly diagnosed FLT3-mut AML in combination with standard chemotherapy
**Gilteritinib**	Type I (selective FLT3 inhibitor)	FLT3-ITD, FLT3-TKD (D835)	AXL ALK LTK	ADMIRAL (R/R AML vs. salvage chemo) MORPHO (post-allo-HCT maintenance) frontline and triplet studies	QTc prolongation; differentiation syndrome; resistance via RAS/MAPK activation	Approved (2018) for relapsed/refractory FLT3-mutated AML
**Quizartinib**	Type II (selective FLT3 inhibitor)	FLT3-ITD (inactive vs. D835)	c-KIT (moderate)	QuANTUM-R (R/R FLT3-ITD AML) QuANTUM-First (frontline FLT3-ITD AML + maintenance)	Ineffective against TKD mutations QTc prolongation cytopenias	Approved (2023) for newly diagnosed FLT3-ITD AML in combination with intensive chemotherapy
**Crenolanib**	Type I (highly selective FLT3 inhibitor)	FLT3-ITD, FLT3-TKD (incl. D835, F691L)	Minimal off-target (weak PDGFR/KIT)	Phase II (R/R AML) ongoing Phase III (NCT03258931) vs. midostaurin (frontline)	GI and hepatic toxicity not yet approved	Not approved (under phase III investigation for FLT3-mut AML)
**Sorafenib**	Type II (multikinase inhibitor)	FLT3-ITD (limited TKD activity)	RAF VEGFR PDGFR KIT	Phase I/II (R/R AML) SORAML (frontline AML, ±FLT3 mut) ALLG AMLM16	Broad off-target activity Cytopenias Limited OS benefit QTc prolongation	Not approved for AML (approved for renal, hepatic, thyroid carcinoma)
